# O01 Why is urinary tract infection so hard to diagnose and treat? Hints from a unique human microtissue model of bladder infection

**DOI:** 10.1093/jacamr/dlad077.001

**Published:** 2023-08-02

**Authors:** Carlos Flores, Nazila Jafari, Jefferson Ling, Amanda Loh, Ramón Garcia Maset, Marloes Hoedek, Richard Leung, Angeline Aw, Ian J White, Raymond Fernando, Jennifer L Rohn

**Affiliations:** Centre for Urological Biology, Division of Medicine, University College London, NW3 2PF London, UK; Centre for Urological Biology, Division of Medicine, University College London, NW3 2PF London, UK; Centre for Urological Biology, Division of Medicine, University College London, NW3 2PF London, UK; Centre for Urological Biology, Division of Medicine, University College London, NW3 2PF London, UK; Centre for Urological Biology, Division of Medicine, University College London, NW3 2PF London, UK; Centre for Urological Biology, Division of Medicine, University College London, NW3 2PF London, UK; Centre for Urological Biology, Division of Medicine, University College London, NW3 2PF London, UK; Centre for Urological Biology, Division of Medicine, University College London, NW3 2PF London, UK; Laboratory for Molecular Cell Biology, University College London, WC1E 6BT London, UK; Centre for Urological Biology, Division of Medicine, University College London, NW3 2PF London, UK; Royal Free London NHS Foundation Trust & Anthony Nolan Laboratories, NW3 2QG London, UK; Centre for Urological Biology, Division of Medicine, University College London, NW3 2PF London, UK

## Abstract

Although urinary tract infection (UTI) appears, on the face of it, to be a simple bacterial infection, upwards of one in four UTI cases recur despite antibiotic therapy. While antimicrobial resistance after empirical treatment undoubtedly accounts for some of these failures, recurrent UTI despite the administration of an appropriate antibiotic, or in the face of prophylactic therapy, remains poorly understood and is likely due to complex and multifactorial aspects of the interaction between the pathogen and its host. In parallel, the role of the healthy uromicrobiome in this process is also far from clear. The vast majority of what little we know about UTI host/pathogen interactions at the urothelial surface has been extrapolated from forced infection experiments in mice, a species that does not naturally experience UTI and whose bladder environment differs significantly from that of humans. For this reason, our lab has been developing alternative, human-cell-based lab models to study the behaviour of both uropathogens and healthy commensals using fully stratified and differentiated urothelial platforms, including lab-on-chip models with flow and stretch parameters. In this talk, I will present recent data putting the most common uropathogens through their paces in our most advanced static model, 3D-UHU. All uropathogens tested, including *Escherichia coli*, *Klebsiella*, *Enterococcus*, *Pseudomonas*, Group B *Streptococcus* and *Proteus*, were able to invade the urothelium to take up residence inside cells, where they would be expected to avoid detection by diagnostic urine tests as well as host neutrophils. Intracellular residence would also foil most oral antibiotics, which are poorly permeant to the urothelial barrier. Some bacteria species, especially *Pseudomonas* and *Proteus*, formed biofilms on the surface of the urothelium, a behaviour that would also help the bacteria to withstand antibiotic treatment. What's more, we observed that some bacteria susceptible to antibiotics in diagnostic media were effectively resistant in the human organoid environment, suggesting that there could be a disconnect between predicted drug and clinical outcome. This phenomenon may be partly explained by our experiments showing that, in some species of uropathogen, the urine environment encourages biofilm formation over the free planktonic state. These experiments highlight diverse infection strategies, shed light on how diagnosis and treatment can fail, and reveal strategies for improving both in the future.

